# Posterior cortical atrophy: a diagnostic challenge in a patient with delayed recognition of progressive visuospatial dysfunction

**DOI:** 10.1007/s10072-026-09338-0

**Published:** 2026-08-31

**Authors:** Monika Gołąb-Janowska, Dariusz Kotlęga, Aleksandra Karwowska, Wioletta Pawlukowska, Mateusz Owsiak, Wojciech Poncyliusz, Małgorzata Poniatowska-Roszkowska, Bożena Birkenfeld

**Affiliations:** 1 Department of Neurology, Pomeranian Medical University, Szczecin, Poland; 2 University Hospitals of Northamptonshire - Northampton General Hospital NHS Trust, Northampton, NN1 5BD UK; 3 Department of Pharmacology and Toxicology, University of Zielona Gora, 65-417 Zielona Gora, Poland; 4 Department of Neurology, University Clinical Hospital No. 1, Pomeranian Medical University in Szczecin, Szczecin, Poland; 5 Department of Radiology and Diagnostic Imaging, Pomeranian Medical University, Szczecin, Poland; 6 Department of Nuclear Medicine, Pomeranian Medical University, Szczecin, Poland

Dear Editor,

Posterior cortical atrophy (PCA) is a rare neurodegenerative syndrome characterized by progressive impairment of higher-order visual processing resulting from predominant involvement of posterior cortical regions. First described by Benson et al., PCA represents a distinct clinical syndrome most commonly associated with Alzheimer’s disease (AD), although other neurodegenerative pathologies, including dementia with Lewy bodies, corticobasal degeneration, and prion diseases, may also underlie this phenotype [1–3]. Due to its atypical clinical presentation, PCA is frequently misdiagnosed as an ophthalmological, psychiatric, or nonspecific cognitive disorder, leading to substantial diagnostic delays [4–6]. We present a patient with clinically and radiologically characteristic PCA whose diagnosis was delayed despite progressive and typical manifestations.

Ethical approval and Informed consent statement: This case report was conducted in accordance with the Declaration of Helsinki. According to the institutional guidelines of the Pomeranian Medical University in Szczecin, formal approval by the Bioethics Committee is not required for the publication of a single case report. Written informed consent was obtained from the patient's legal representative for publication of this case report and the accompanying imaging data (Figure 1). A copy of the written consent is available for review by the Editor-in-Chief of this journal upon request.

A 59-year-old right-handed woman was referred to the Department of Neurology in 2022 because of gradually progressive cognitive and visual disturbances. The symptoms had developed over several years and initially included memory complaints, visuospatial difficulties, mood changes, and psychomotor slowing. Before establishing the final diagnosis, the patient underwent repeated psychiatric and neurological evaluations, during which conversion disorder, organic mood disorder, and unspecified dementia were considered.

Her medical history included arterial hypertension, dyslipidemia, migraine with visual aura, and corrected refractive error. Neurological and neuropsychological examination revealed impairment of recent memory and visuospatial orientation, together with clinical features characteristic of posterior cortical dysfunction. The patient demonstrated elements of Bálint syndrome, including simultanagnosia, optic ataxia, and ocular apraxia, as well as features of Gerstmann syndrome, including agraphia, acalculia, right–left disorientation, and finger agnosia. A right homonymous hemianopia and mild parkinsonian features, including bradykinesia and increased muscle tone, were also observed. The Mini-Mental State Examination score was 16 points, and the clock-drawing test score was 2 points [4, 7].

Brain magnetic resonance imaging (MRI) demonstrated predominant parieto-occipital cortical atrophy with widening of posterior cingulate and parieto-occipital sulci, more pronounced on the left side. Brain perfusion scintigraphy with [99mTc]Tc-HMPAO revealed marked hypoperfusion of the parietal and occipital cortical regions, also with left-sided predominance (Fig. 1). These findings corresponded to the characteristic posterior cortical pattern observed in PCA and supported the clinical diagnosis [4, 5, 8]. Routine laboratory investigations and cerebrospinal fluid analysis were unremarkable, and AD-specific biomarkers were not available.

**Fig. 1:**
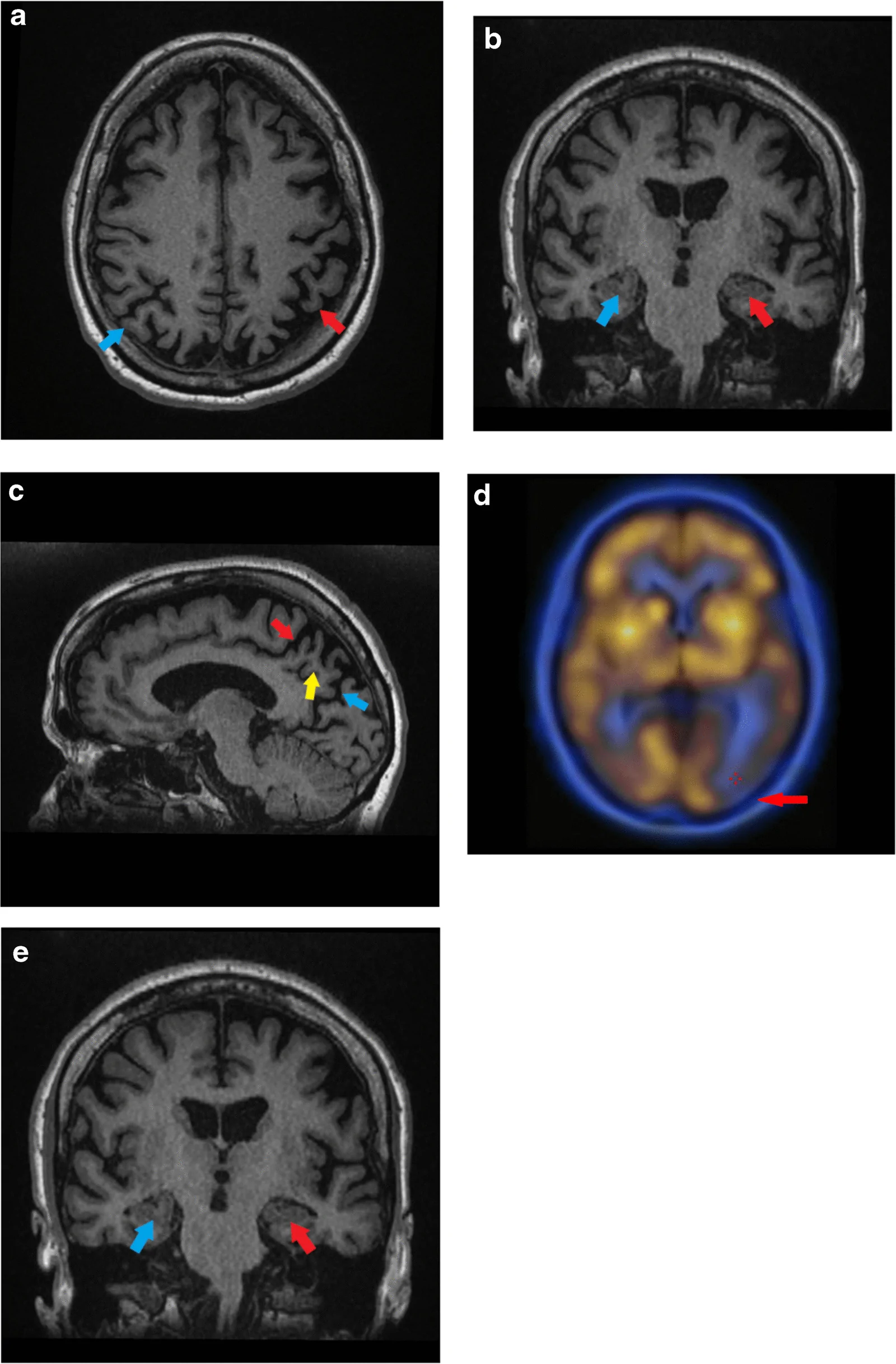

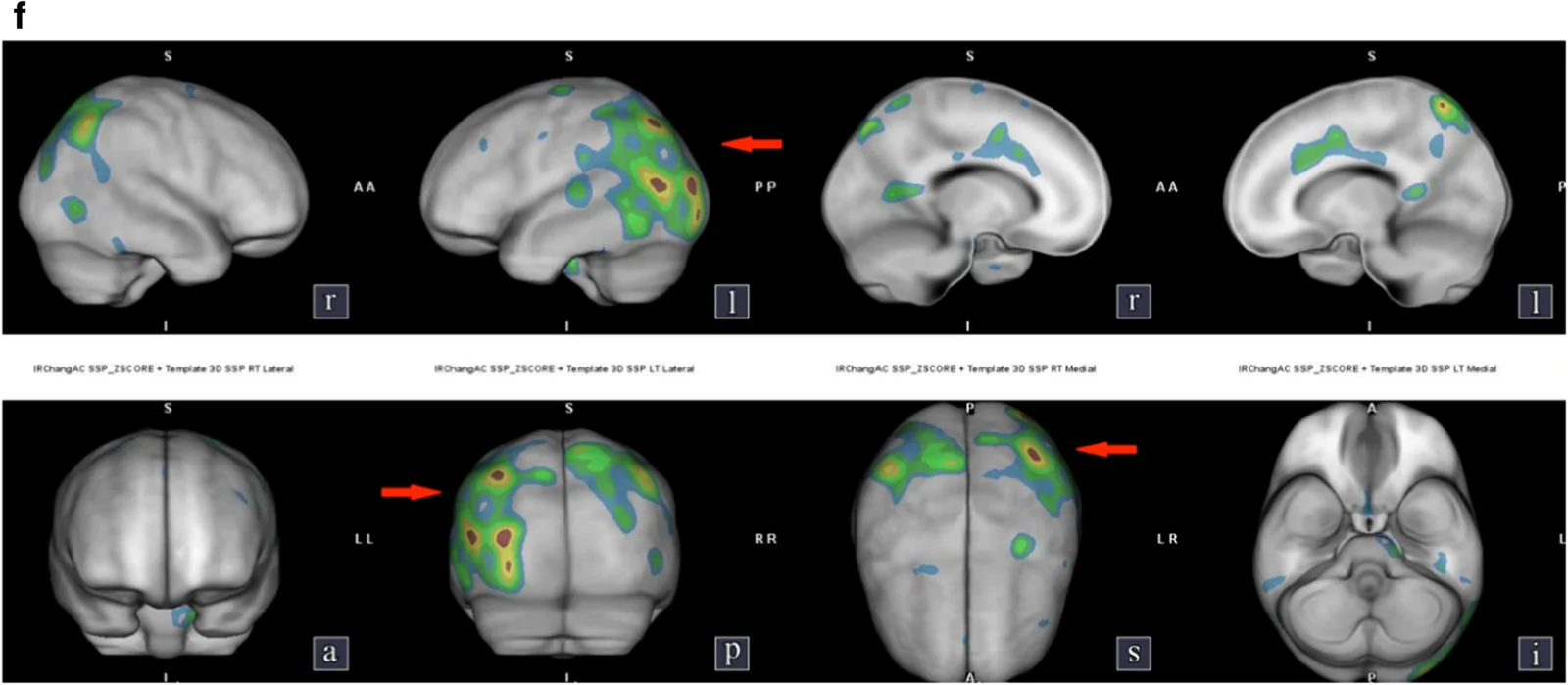
Neuroimaging findings in a patient with posterior cortical atrophy. **A**–**C**. Brain MRI findings: **A**: axial T1-weighted image showing posterior predominant cortical atrophy with parieto-occipital involvement. **B**: coronal T1-weighted image demonstrating widening of posterior cingulate and parieto-occipital sulci. **C**: sagittal image illustrating posterior cortical volume loss. **D**–**F**. Brain perfusion SPECT findings: **D**: axial perfusion image showing reduced tracer uptake in the parietal and occipital cortices. **E**: coronal image demonstrating posterior hypoperfusion with left-sided predominance. **F**: statistical analysis/Q Brain map showing reduced perfusion relative to reference population

Based on the clinical presentation and neuroimaging findings, the patient was diagnosed with pure PCA according to the consensus criteria proposed by Crutch et al. [7]. Treatment with donepezil and memantine was initiated, and cognitive rehabilitation was recommended.

The presented case highlights several important diagnostic challenges associated with PCA. Although the patient fulfilled the clinical and radiological criteria for PCA at the time of neurological assessment, the diagnosis was delayed for several years. This reflects a common problem in PCA, as early symptoms are often dominated by visuospatial and visuoperceptual disturbances rather than typical episodic memory impairment. Consequently, patients may initially be evaluated for ophthalmological disorders, psychiatric conditions, or nonspecific cognitive complaints before a neurodegenerative cause is recognized [4–6].

The clinical manifestations observed in our patient were highly characteristic of posterior cortical dysfunction. The presence of Bálint syndrome, Gerstmann syndrome, visuospatial impairment, and homonymous hemianopia represents a typical constellation of findings associated with PCA [4, 7]. Importantly, these symptoms may be overlooked if cognitive assessment focuses predominantly on memory and language domains. Detailed evaluation of higher-order visual functions is therefore essential in patients presenting with unexplained visual complaints or atypical cognitive decline.

Neuroimaging played a crucial role in establishing the diagnosis. The characteristic pattern of posterior cortical atrophy on MRI, together with marked parieto-occipital hypoperfusion on [99mTc]Tc-HMPAO SPECT, supported the diagnosis of PCA [4, 5, 8]. Although PCA represents a distinct clinical syndrome, neuropathological and biomarker studies indicate that AD is the most frequent underlying pathology [2, 3, 6, 9]. The absence of AD-specific biomarkers in our patient represents a limitation; however, current diagnostic criteria allow a clinicoradiological diagnosis of PCA when characteristic clinical and imaging features are present [7].

The presence of mild parkinsonian features initially broadened the differential diagnosis and raised consideration of dementia with Lewy bodies and corticobasal syndrome. However, the absence of core clinical features of these disorders, including cognitive fluctuations, recurrent visual hallucinations, REM sleep behavior disorder, marked asymmetric motor dysfunction, limb apraxia, or cortical sensory deficits, made these diagnoses less likely [10, 11]. Similarly, the slowly progressive course and lack of rapid neurological deterioration argued against prion disease [4].

This case emphasizes the importance of recognizing PCA as a distinct neurodegenerative syndrome and considering it in patients with progressive visuospatial impairment, even when memory dysfunction or psychiatric symptoms predominate. Early identification of characteristic clinical signs and posterior cortical abnormalities on neuroimaging may reduce diagnostic delays and prevent unnecessary investigations. Although disease-modifying therapy is currently unavailable, timely diagnosis enables appropriate symptomatic treatment, cognitive rehabilitation, patient counseling, and long-term care planning.

In conclusion, increased awareness of PCA among neurologists and other clinicians involved in the evaluation of cognitive and visual symptoms is essential to improve diagnostic accuracy. Careful assessment of higher-order visual functions combined with appropriate neuroimaging may facilitate earlier recognition of this underdiagnosed but clinically distinctive syndrome [4, 7].

## Data Availability

Data sharing is not applicable to this article as no datasets were generated or analyzed during the current study.
